# Inhibition of Ceramide De Novo Synthesis Affects Adipocytokine Secretion and Improves Systemic and Adipose Tissue Insulin Sensitivity

**DOI:** 10.3390/ijms19123995

**Published:** 2018-12-11

**Authors:** Agnieszka U. Blachnio-Zabielska, Hady Razak Hady, Adam R. Markowski, Adam Kurianiuk, Alicja Karwowska, Jan Górski, Piotr Zabielski

**Affiliations:** 1 Department of Hygiene, Epidemiology and Metabolic Disorders, Medical University of Bialystok, 15-089 Bialystok, Poland; adam.kurianiuk@umb.edu.pl (A.K.); alicja.karwowska@umb.edu.pl (A.K.); 2 Department of Physiology, Medical University of Bialystok, 15-089 Bialystok, Poland; gorski@umb.edu.pl (J.G.); piotr.zabielski@umb.edu.pl (P.Z.); 3 1st Department of General Surgery and Endocrinology, Medical University Bialystok, 15-276 Bialystok, Poland; hadyrazakh@wp.pl; 4 Department of Internal Medicine and Gastroenterology, Polish Red Cross Memorial Municipal Hospital, 15-003 Bialystok, Poland; adromax@wp.pl; 5 Department of Basic Sciences, Faculty of Health Sciences, Lomza State University of Applied Sciences, 18-400 Lomza, Poland; 6 Department of Medical Biology, Medical University of Bialystok, 15-089 Bialystok, Poland

**Keywords:** ceramide, insulin resistance, visceral adipose tissue, subcutaneous adipose tissue, metabolic disorders, mass spectrometry

## Abstract

Ceramide accumulation in muscle and in liver is implicated in the induction of insulin resistance. Much less in known about the role of ceramide in adipose tissue. The aim of the present study was to elucidate the role of ceramide in adipose tissue and to clarify whether lipids participate in the regulation of adipocytokine secretion. The experiments were performed on male Wistar rats divided into three groups: 1. Control, 2. fed high fat diet (HFD), and 3. fed HFD and treated with myriocin. Ceramide (Cer) and diacylglycerol (DAG) content were analyzed by LC/MS/MS. Hormone sensitive lipase (HSL) phosphorylation was analyzed by Western Blot. Plasma adiponectin and tumor necrosis factor alpha (TNF-α) concentration were measured by enzyme-linked immunosorbent assay. An oral glucose tolerance test (OGTT) and insulin tolerance test (ITT) was also performed. In HFD group, total DAG and Cer content was elevated in both subcutaneous and visceral adipose tissue, which was accompanied by increased glucose, insulin, and HOMA-IR value. Myriocin treatment restored HOMA-IR as well as glucose and insulin concentration to control values. Moreover, myriocin decreased not only Cer, but also DAG levels in both fat depots. Furthermore, we observed a strong correlation between adiponectin (negative) and TNF-α (positive) and Cer in both fat tissues, which suggests that Cer is involved in the regulation of adipocytokine secretion.

## 1. Introduction

Adipose tissue plays an important role in the induction of insulin resistance associated with obesity. The basic role of this tissue is to store fatty acids in the form of triacylglycerols as an energy reserve. However, adipose tissue also plays an important endocrine function. In the tissue, adipocytokines are synthesized and secreted into the bloodstream, which affects the insulin sensitivity of other tissues, such as skeletal muscles and the liver [1,2]. Among these compounds are: leptin, adiponectin, tumor necrosis factor-α (TNF-α), interleukin-6 (IL-6), and plasminogen activator inhibitor-1 (PAI-1) [3,4,5]. It is well documented that adiponectin affects lipid and glucose metabolism through adenosine monophosphate-activated protein kinase (AMPK) phosphorylation [6]. Activated AMPK stimulates phosphorylation of acetylCoA carboxylase (ACC - key enzyme in fatty acids de novo synthesis) as well as fatty acid oxidation and glucose uptake in muscle. Moreover, AMPK inhibits enzymes involved in gluconeogenesis in liver, which leads to a reduction of plasma glucose concentration [6]. In addition, obesity is accompanied by moderate inflammation, in which adipocytes and immune cells residing in adipose tissue contribute to increasing the level of circulating proinflammatory cytokines. One such cytokine that is involved in the inflammatory reaction that links central obesity with insulin resistance is TNF-α [7]. TNF-α has been shown to inhibit insulin-stimulated tyrosine kinase activity of the insulin receptor as well as insulin receptor substrate 1 (IRS-1), by inducing a serine phosphorylation of IRS-1 [8]. Furthermore, it is known that TNF-α activates sphingomyelinase, thereby promoting the production of ceramide [9].

Fat tissue is located in numerous parts of an organism. The tissues from different depots have distinct metabolic activities. Abdominal obesity correlates most strongly with metabolic disorders including insulin resistance [10,11,12]. Abdominal obesity is associated with an excess of not only subcutaneous fat (SAT), but also visceral fat (VAT). Visceral tissue is metabolically a very active tissue and easily liberates fatty acids. Therefore, in terms of the induction of insulin resistance, the attention of researchers focuses on metabolic disorders occurring in this tissue. Under physiological conditions, insulin inhibits the activity of hormone sensitive lipase (HSL), so in the presence of high glucose concentration, the release of fatty acids is limited. In an insulin resistant state, insulin is not able to inhibit HSL, which results in an increased plasma free fatty acid (FFA) concentration. Plasma FFAs are uptaken by other peripheral tissues, where they are used in the β-oxidation as an energy source or as a substrate for de novo synthesis of other lipids. It has been demonstrated several times that lipid accumulation in skeletal muscle or in the liver is responsible for the induction of insulin resistance [13,14]. Among these lipids are ceramides (Cer) and diacylglycerols (DAG). The content of ceramide inside a cell is determined by a balance between the rate of its production and the rate of its degradation. There are two main types of ceramide formation. The first way is through hydrolysis of sphingomyelin, which is located in numerous places inside a cell and is hydrolyzed by the neutral and acid sphingomyelinase (n- and aSMase). The second type of ceramide production is de novo biosynthesis. The first, and rate-limiting step of de novo ceramide synthesis, is condensation of serine with palmitoyl-CoA leading to the formation of 3-ketosphinganine. This reaction is catalyzed by the enzyme serine-palmitoyltransferase (SPT). 3-ketosphingosine is reduced to sphinganine (SPA) by the enzyme 3-ketosphinganine reductase. Next, SPA is acylated to form dihydroceramide by the action of ceramide synthase. The last step of ceramide synthesis is conversion of dihydroceramide to ceramide by the enzyme dihydroceramide desaturase [15]. Initially, the contribution of lipid accumulation in the induction of insulin resistance was observed in skeletal muscle [16,17,18]. Subsequently, the effect of Cer and DAG accumulation in induction of insulin resistance was noticed in the liver [19,20,21,22]. In both tissues it has been shown that lipids inhibit the insulin signaling pathway. Much less is known about the effect of Cer and DAG accumulation in adipose tissue. There are only a few papers indicating the relationship between the accumulation of these lipids and the IRes [23,24,25,26,27,28]. The existing data indicate that ceramide content is higher in both the SAT and epicardial adipose tissue (EAT) of obese people, as compared to lean counterparts. In addition, a positive correlation between C16:0-Cer and HOMA-IR, and between HOMA-IR and C16:0/18:2 DAG in SAT was noticed [24,25]. Myriocin is a fungal metabolite that inhibits SPT activity, blocking the synthesis of ceramide. Because adipose tissue plays a superior role in the induction of insulin resistance and ceramide accumulation affects the insulin action in other tissues, it seems that studying the role of ceramide accumulation in different fat depots on insulin sensitivity is important. Therefore, we designed the present study to examine the effect of a high fat diet (HFD) on the content of ceramide and DAG in visceral and subcutaneous fat tissues. Moreover, because ceramide has begun to be perceived as a dominant agent inducing insulin resistance, we tried to explain the role of ceramide in the induction of metabolic disorders at the level of adipose tissue by inhibiting ceramide de novo synthesis. Our goal was also to clarify whether biologically-active lipids participate in the regulation of adipocytokine secretion by checking the correlation between the content of these lipids in both fat tissues and adiponectin and TNF-α concentration in plasma.

## 2. Results

### 2.1. HOMA-IR, OGTT and ITT

**Figure 1:**
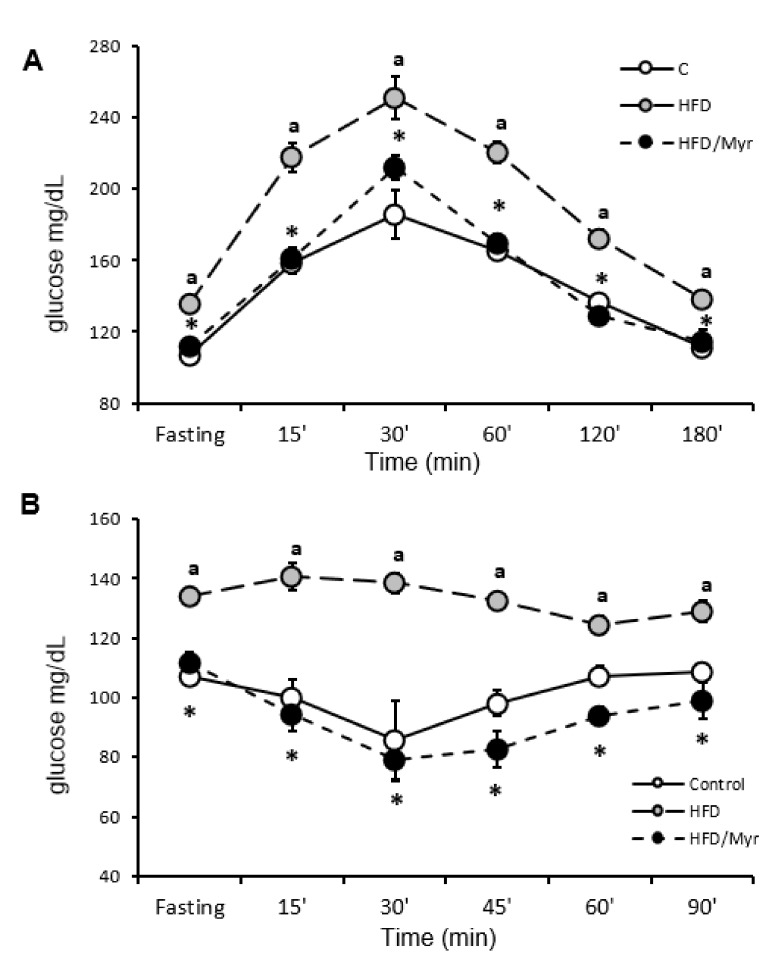
Myriocin treatment improved glucose and insulin tolerance in animals on a high-fat diet. Panel (**A**) shows blood glucose profiles obtained during oral glucose tolerance test (OGTT); Panel (**B**) shows blood glucose profiles during intraperitoneal insulin tolerance test. Values are mean +/− SD. Symbols denote statistical significance of *p* < 0.05 against: ^a^—vs. control group; *—vs. HFD group.

Animals fed HFD developed IRes, as evidenced by elevated fasting blood glucose concentration (Table 1), impaired glucose tolerance (Figure 1A), reduced insulin responsiveness (Figure 1B), and increased HOMA-IR index. HOMA-IR index increased by 46% (*p* < 0.05) in HFD group compared to the control group. Myriocin normalized insulin-related parameters to control values (Table 1).

### 2.2. Plasma FFAs

Total plasma FFA concentration increased in HFD and HFD/Myr groups by 30% and 45%, respectively (*p* < 0.05), as compared to control group (Table 1). In HFD group, the highest increases were noticed in long-chain saturated FAs: C18:0, C20:0, and C24:0. Moreover, we observed a decrease in unsaturated FA content (C16:1 and C18:1). In the HFD/Myr group, the highest increases were observed in C24:1, C22:0, C20:0, C18:0, C16:0, and C18:2 (Table 2).

### 2.3. Plasma Adiponectin and TNF-α Concentration

Plasma adiponectin concentration significantly decreased in HFD group as compared to control group (*p* < 0.05). Myriocin treatment restored adiponectin concentration to the control value. Plasma TNF-α concentration increased in HFD group as compared to control group (*p* < 0.05), whereas myriocin treatment caused a decrease in adiponectin concentration as compared to HFD group (*p* < 0.05) (Table 1).

### 2.4. Hormone Sensitive Lipase pHSLSer563

**Figure 2:**
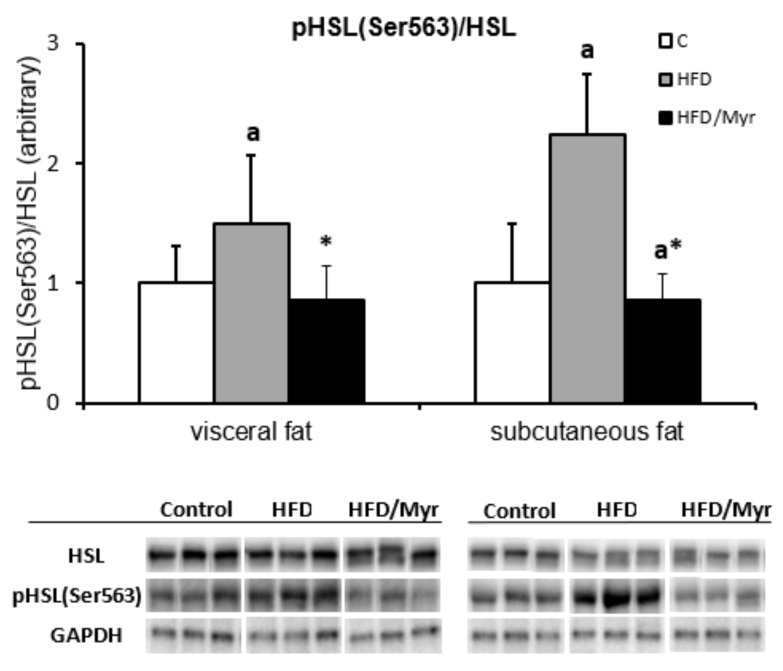
A ratio of pHSL (Ser563) to HSL in two depots of adipose tissue. White bars—control group; grey bars—HFD group; black bars—HFD/Met group. Values are mean +/− SD. Symbols denote statistical significance of *p* < 0.05 versus: ^a^—vs. Control group; *—vs. HFD group.

The phosphorylation of the enzyme at the Ser563 residue increased in both fat depots in the HFD group. In the group treated with myriocin, the phosphorylation of HSL decreased in both fat depots as compared to HFD group (*p* < 0.05) (Figure 2).

### 2.5. Ceramide and DAG Concentration

**Figure 3:**
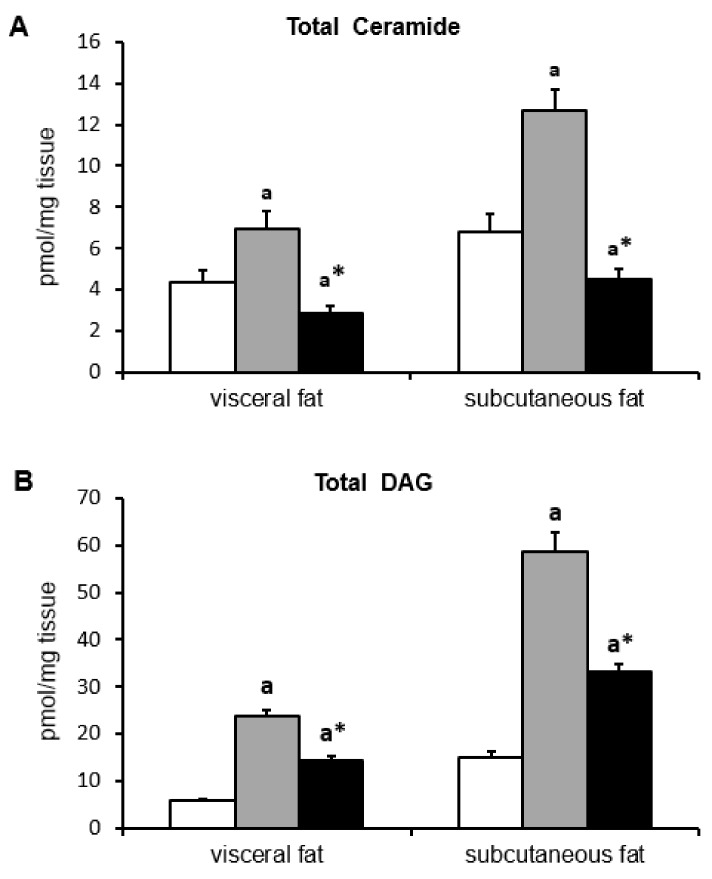
Panel (**A**) presents total ceramide content in two depots of adipose tissue. Panel (**B**) shows total DAG content in two depots of adipose tissue. White bars—control group; grey bars—HFD group; black bars—HFD/Myr group. Values are mean +/− SD. Symbols denote statistical significance of *p* < 0.05 versus: ^a^—vs. control group; *—vs. HFD group.

In the group fed HFD, total content of ceramide (Figure 3A) and DAG (Figure 3B) significantly increased in subcutaneous and visceral fat tissue as compared to control group. Myriocin treatment reduced total content of these lipids as compared to HFD group (for all *p* <0.05), however, the content of ceramide in the group was significantly lower than in control group. In visceral adipose tissue, the highest elevation in HFD group was observed in C18:1-Cer and C18:0-Cer (*p* < 0.05) (Table 3). In the same group, the largest increase of ceramide content in subcutaneous adipose tissue was observed in C18:1-Cer, C22:0-Cer, and C24:0-Cer. Myriocin treatment caused a significant decrease in all measured ceramides in both fat depots as compared to both HFD group and control group. The only exception is C18:1-Cer in subcutaneous fat, where its level did not differ from its content in the control group (*p* < 0.05) (Table 3).

In HFD group, the level of all measured DAG was significantly higher in both depots of adipose tissue as compared to control group (*p* < 0.05). In the group fed HFD and treated with myriocin, the content of all DAG was lower than in the HFD group, but higher than in the control group, with the exception of 16/16 in the subcutaneous group, where its content did not differ from its content in the HFD group (Table 4).

### 2.6. Correlation between Ceramide and DAG with HOMA-IR, Adiponectin, and TNF-α

**Figure 4:**
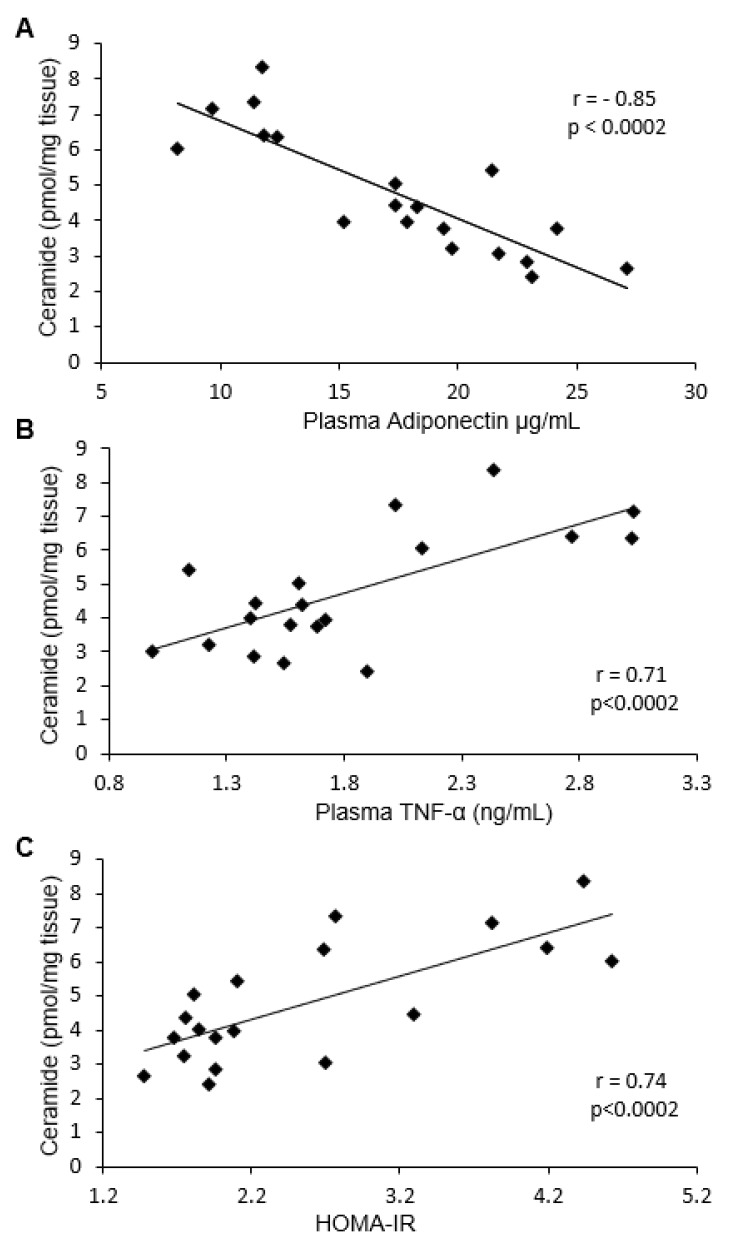
Panel (**A**) shows the correlation between ceramide content in visceral tissue and plasma adiponectin concentration. Panel (**B**) shows the correlation between ceramide content in visceral tissue and plasma TNF-α concentration. Panel (**C**) shows the correlation between ceramide content in visceral tissue and HOMA-IR value.

**Figure 5:**
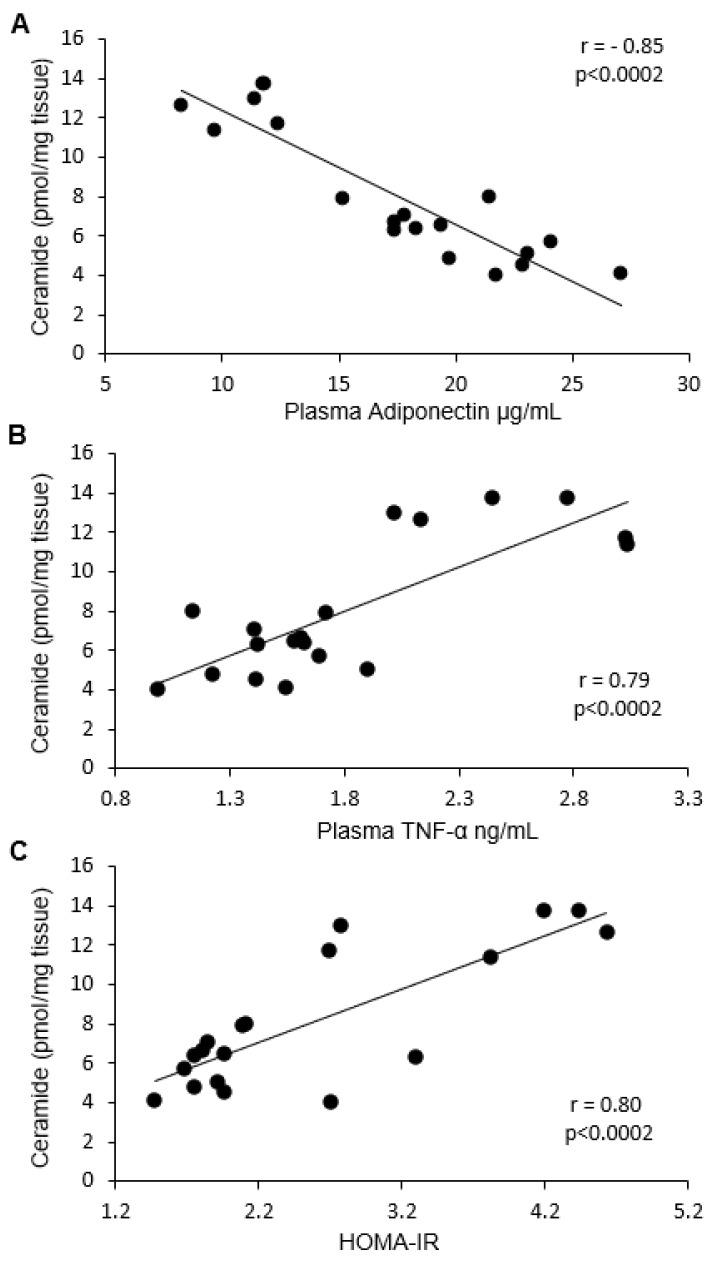
Panel (**A**) shows the correlation between ceramide content in subcutaneous fat tissue and plasma adiponectin concentration. Panel (**B**) shows the correlation between ceramide content in subcutaneous fat tissue and plasma TNF-α concentration. Panel (**C**) shows the correlation between ceramide content in subcutaneous fat and HOMA-IR value.

We noticed a strong negative correlation between total ceramide content in both visceral and subcutaneous fat tissue and adiponectin concentration (Pearson’s *r* = −0.85 in both cases) (Figure 4A and Figure 5A). In terms of particular ceramide species, the strongest negative correlation was observed for C18:1-Cer, C18-Cer, C20-Cer, C22-Cer, and C24-Cer in visceral tissue, and for C16-Cer, C18:1-Cer, C18-Cer, C20-Cer, C22-Cer, and C24-Cer in subcutaneous fat tissue. Moreover, a strong, positive correlation was found between plasma TNF-α concentration and C18:1-Cer, C18-Cer, C20-Cer, and total ceramide in visceral adipose tissue (Pearson’s *r* = 0.71) (Figure 4B), as well as between TNF-α concentration and C16-Cer, C18:1-Cer, C18-Cer, C24-Cer, and total ceramide level in subcutaneous fat tissue (Pearson’s *r* = 0.79) (Figure 5B). Furthermore, we found a positive correlation between HOMA-IR value and visceral fat tissue C18:1-Cer, C18-Cer, C20-Cer, and total ceramide content (Pearson’s *r* = 0.74) (Figure 4C), as well as between HOMA-IR and subcutaneous adipose tissue C16-Cer, C18-Cer, C22-Cer, C24-Cer, and total Cer level (Pearson’s *r* = 0.8) (Figure 5C). 

In the case of adiponectin and DAG, the only strong negative correlation was observed with 18/18:1 DAG in subcutaneous adipose tissue (Pearson’s *r* = −0.78). Moreover, plasma TNF-α concentration positively correlated with all measured DAG species in subcutaneous fat tissue (Pearson’s *r* > 0.72 in all cases) (except 16/16). In addition, a strong, positive correlation between the HOMA-IR value and visceral adipose tissue 18/18:1, as well as subcutaneous fat tissue 16/18:2, 18/18:1, and total DAG content was also observed (Pearson’s *r* > 0.82 in all cases).

## 3. Discussion

In the present study, we found that inhibition of ceramide de novo production by myriocin treatment reversed insulin resistance induced by HFD, which was manifested by lower HOMA-IR value and by improvement of glucose and insulin tolerance. Adipose tissue, besides skeletal muscle and the liver, is the main tissue responsible for insulin-dependent glucose metabolism, therefore, metabolic disturbances in these tissues lead to the induction of insulin resistance. Adipose tissue insulin resistance is mainly related to the inability of insulin to inhibit HSL activity. In a physiological state, insulin inhibits HSL activity, and, therefore, the release of FFA from TAG is reduced. However, in the IRes state, insulin is not able to effectively inhibit HSL activity and plasma FFA levels increase. The HSL activation is related to phosphorylation of the enzyme in numerous serine residues, including Ser-563. In the present work, we found that in the group fed HFD, HSL phosphorylation at Ser-563 increased in both fat depots, which explains the increase in plasma FFA levels. However, in the myriocin treated group, where we observed an improvement in insulin sensitivity, we also noticed a decrease in HSL phosphorylation at Ser-563 in visceral and subcutaneous fat tissue, which suggests that enzyme activity was inhibited. The data indicate a beneficial effect of myriocin, not only systemic, but also on fat tissue insulin sensitivity. Typically, insulin resistance is associated with increased plasma FFA concentration [29,30,31,32,33,34,35,36], and improvement of insulin sensitivity is accompanied by a decrease in plasma FFA levels. As expected, in the group fed HFD we noticed increased plasma FFA concentration, especially long-chain fatty acids. Surprisingly, in the group fed HFD and treated with myriocin, although we did notice an improvement in insulin sensitivity, we did not observe a decrease, rather an even more significant increase of FFA concentration in comparison to HFD group. It is even more interesting that in the myriocin-treated group, plasma FFAs are elevated even though the activity of HSL is inhibited as compared to HFD group. Our previously published works regarding the role of the inhibition of ceramide production on skeletal muscle and liver metabolism have demonstrated that in skeletal muscle [37] and in the liver [38], the uptake of fatty acids are deteriorated, which explain the elevated plasma FFA concentration. In the present study, we tried to explain how the inhibition of ceramide synthesis affects lipid composition in both visceral and subcutaneous fat tissue, and how the changes affect adipocytokine secretion. Adipocytokines affect the insulin sensitivity of other tissue, such as skeletal muscle or the liver. It has been repeatedly reported that accumulation of biologically active lipids, ceramide, and DAG in skeletal muscle and in the liver is associated with IRes [16,17,18,37,38,39,40,41,42]. In our work, we have demonstrated that HFD led to the accumulation of ceramide and DAG in both depots of fat tissue. This was associated with adipose tissue insulin resistance which was visualized by an increase of HSL phosphorylation. Myriocin treatment of animals fed HFD caused a decrease in both ceramide and DAG in visceral and subcutaneous fat tissue. Myriocin is known to inhibit SPT activity, and in this way inhibits ceramide production. Therefore, the decrease in ceramide content in the group is not surprising. More enigmatic is the decreased content of DAG. We suggest that the lower DAG content is an effect of limited fatty acids uptake, as it was noticed in skeletal muscle and in the liver [37,38]. The role of bioactive lipid accumulation in adipose tissue is very poorly understood. There are only few papers demonstrating that in obesity, ceramide content is elevated in subcutaneous adipose tissue [24,25,43]. An increased content of both ceramide and DAG in obesity was observed in epicardial fat tissue [25]. Adipose tissue not only serves to store fatty acids in the form of triacylglycerols, but also performs an important endocrine function, secreting a number of biologically active compounds named adipocytokines. Among these compounds are many that affect the insulin sensitivity of other tissues, such as skeletal muscle and the liver. In the present work, we analyzed the adiponectin and TNF-α concentration and the correlation between these adipocytokines and bioactive lipids in both fat depots. We have demonstrated that TNF-α increased while adiponectin decreased in the group fed HFD. Myriocin treatment restored the adiponectin and TNF-α concentration to control value. Moreover, we noticed a strong, negative correlation between plasma adiponectin concentration and C18:1-Cer, C18-Cer, C20-Cer, C22-Cer, C24-Cer, and total Cer in visceral adipose tissue, and between plasma adiponectin and C16-Cer, C18:1-Cer, C18-Cer, C20-Cer, C22-Cer, C24-Cer, and total Cer in subcutaneous fat tissue. In subcutaneous adipose tissue, a negative correlation between C16-Cer and plasma adiponectin was previously demonstrated in women [24]. The presented results demonstrate that ceramide accumulation in adipose tissue inhibits adiponectin secretion from adipose tissue. On the other hand, we have also noticed a strong, positive correlation between plasma TNF-α concentration and C18:1-Cer, C18-Cer, C20-Cer, and total Cer in visceral adipose tissue, and between TNF-α concentration and C16-Cer, C18:1-Cer, C18-Cer, C24-Cer, and total Cer in subcutaneous fat tissue. These data indicate that ceramide accumulation in both types of adipose tissue negatively affects insulin sensitivity by inhibiting the release of adiponectin and increasing the secretion of TNF-α.

In summary, we have demonstrated that HFD induced IRes, which was visualized by increased HOMA-IR value as well as glucose and insulin concentration. In addition, in the group fed HFD we observed an increased content of Cer and DAG in both fat depots as well as an increased concentration of TNF-α and FFA and decreased plasma adiponectin concentration. Moreover, HFD led to an increase in HSL phosphorylation. However, inhibition of ceramide de novo synthesis restored HOMA-IR value as well as insulin and glucose concentration. In the myriocin treated group, we have also noticed a return to control values of adiponectin and TNF levels. In this work, we have shown for the first time that in vivo, myriocin caused a decrease in Cer and DAG levels in visceral and subcutaneous fat tissue as well as inhibition of HSL activity. Furthermore, we observed a strong correlation between adiponectin (negative) and TNF-α (positive) and the content of ceramide in the both fat tissues. Therefore, we postulate that ceramide accumulation that accompanies obesity affects adipocytokine secretion.

## 4. Materials and Methods

### 4.1. Animals and Study Design

The investigation was approved by the Institutional Animal Care and Use Committee of Medical University of Bialystok (approval no. 2011/49, 26 October 2011). All methods were performed in accordance with the relevant guidelines and regulations. The experiments were performed on male Wistar rats (140–150g) housed in standard conditions (21 ± 2 °C, 12 h light/12 h dark cycle) with free access to tap water and food pellets. Animals were divided into the following groups (*n* = 8 in each group): (1) control group, fed ad libitum a control diet (Research Diets INC D12450B, Research Diets, New Brunswick, NJ), (2) group fed HFD (Research Diets INC D12492), and (3) myriocin-treated (HFD/Myr) group (*n* = 7) fed HFD with daily intraperitoneal injection of SPT inhibitor myriocin (0.5 mg/kg, in PBS buffer). The control diet contained 10% kcal from fat while the HFD contained 60% kcal from fat. All groups were fed for 8 weeks with their appropriate diet. One day before the animals were sacrificed, an oral glucose tolerance test (OGTT) and an insulin tolerance test (ITT) were undertaken. Fasting plasma insulin and glucose concentration was measured for HOMA-IR calculation. Half an hour before sacrifice, insulin (0.5 U/kg) was administrated. The rats were anaesthetized by intraperitoneal injection of pentobarbital in a dose of 80 mg/kg of body weight. The fat tissue from two depots was taken and frozen in liquid nitrogen and then stored at −80 °C until analysis.

### 4.2. Lipids Measurements

Plasma FFA concentration was measured by UHPLC/MS (Agilent, Santa Clara, CA, USA) according to Persson et al. [44]. Fatty acids were separated on the LC using a reverse-phase Zorbax SB-C18 column 2.1 × 150 mm, 1.8 µm (Agilent, Santa Clara, CA, USA), using two buffers. Buffer A was 80% acetonitrile, 0.5 mM ammonium acetate; buffer B was 99% acetonitrile, 1% 0.5 mM ammonium acetate.

Ceramide content in both fat depots was analyzed with the use of an UHPLC/MS/MS (Agilent, Santa Clara, CA, USA) approach according to Blachnio-Zabielska et al., with minor modifications [45]. The adipose tissue samples (~20 mg) were briefly pulverized and after that homogenized in a solution composed of 0.25 M sucrose, 25 mM KCl, 50 mM Tris, and 0.5 mM EDTA, pH 7.4. Immediately afterwards, the internal standard (d17:1/8:0, d17:1/18:0, d17:1/18:1(9Z), d17:1/20:0, d17:1/24:0, and d17:1/24:1(15Z) (Avanti Polar Lipids, Alabaster, AL, USA) as well as extraction mixture (isopropanol:water:ethyl acetate; 30:10:60; *v*:*v*:*v*) were added to each homogenate. The mixture was vortexed, sonicated, and then centrifuged for 10 min at 4000 rpm. The supernatant was transferred to a new tube and the pellet was re-extracted. Supernatants were combined and evaporated under nitrogen. The dried sample was reconstituted in 100µL of LC Solvent A (2 mM ammonium formate, 0.15% formic acid in methanol) for LC/MS/MS analysis. Measurements were made using a triple quadrupole mass spectrometer operated in positive ion electrospray ionization (ESI) with multiple reaction monitoring (MRM). Concentration of each compound was analyzed against the concentration standard curves.

The content of DAG was measured using a UHPLC/MS/MS approach according to Blachnio-Zabielska et al. [46]. Diacylglycerols were extracted together with sphingolipids. A known amount of internal standard mix (Deuterated DAG Mixture I and Mixture II—Avanti Polar Lipids) was added to each sample. Next, samples were extracted as described above. The following DAG species were quantified: C18:1/18:2, C16:0/18:2, C16:0/16:0, C16:0/18:1, C18:0/20:0, C18:0/18:1, C18:1/18:1, C18:0/18:2, and C16:0/18:0 using UHPLC/MS/MS. Diacylglycerols content were analyzed by means of a triple quadrupole mass spectrometer using positive ion electrospray ionization (ESI) source with multiple reaction monitoring (MRM) against the concentration standard curves.

### 4.3. Western Blot

Equal amounts of protein (20 mg) were separated by 10% SDS–PAGE. Separated proteins were transferred on PVDF membranes. The membrane was probed with an appropriate primary antibody. The following target proteins were quantified using primary antibodies HSL and pHSL (Ser563) (Cell Signaling, Danvers, MA, USA). Values were normalized to GAPDH protein expression measured from parallel runs and expressed as fold changes over control group values. Unless stated otherwise, all chemicals and equipment used for immunoblotting were purchased from Bio-Rad (Hercules, CA, USA).

### 4.4. Plasma Adiponectin and TNF-α Concentration 

The plasma adiponectin and TNF-α concentration were measured using commercially available enzyme-linked immunosorbent assay ELISA kits (Abcam, Cambridge, MA, USA), according to the manufacturer’s instructions.

### 4.5. Oral Glucose Tolerance Test (OGTT)

Blood samples from tail veins were collected in fasted animals 15, 30, 60, 120, and 180 min after oral glucose administration in a dose of 3 g/kg. Blood glucose was measured using a glucometer AccuChek (Roche, Germany).

### 4.6. Insulin Tolerance Test (ITT)

Fasted animals received intravenous an injection of insulin in a dose of 0.75 U/kg body weight. Glucose concentration was measured in blood obtained from the tail vein at 0, 15, 30, 45, 60, and 90 min after insulin injection with the use of an AccuChek glucometer (Roche, Germany).

### 4.7. Plasma Insulin and Glucose Concentration

Plasma glucose concentration was measured using an AccuChek glucometer (Roche, Germany). Plasma insulin concentration was determined with an ELISA insulin assay (Rat/Mouse Insulin ELISA Kit, Millipore, Burlington, MA, USA).

### 4.8. HOMA-IR

HOMA-IR index value was calculated according to formula [47]:

HOMA-IR = [fasting glucose (mg/dL) × fasting insulin (lU/mL)]/2430

### 4.9. Protein Concentration

Protein concentration in homogenates was measured with the BCA protein assay kit (Sigma-Aldrich, Saint Louis, MO, USA). Bovine serum albumin (fatty acid free) was used as a standard.

### 4.10. Statistical Significance Estimation and Correlation Analysis

Statistical significance between experimental groups was estimated using ANOVA with Tukey HSD post-hoc test for unequal n-numbers. Significance level was set to *p* < 0.05. We used Pearson’s r approach with Bonferroni correction for multiple comparisons to establish relationships between selected variables. For adipokines and insulin sensitivity parameters Pearson’s r *p*-value was adjusted to 0.002 (25 correlations). For adipokines and individual molecular species of adipose tissue lipids, Pearson’s r *p*-value was adjusted to 0.0002 (249 correlations).

## 5. Conclusions

In this study, we have demonstrated for the first time that in vivo myriocin treatment results in a reduction of the level of Cer and DAG in visceral and subcutaneous adipose tissue, as well as inhibition of HSL activity. Furthermore, we observed a strong correlation between adiponectin (negative) and TNF-α (positive) and the content of ceramide in the both fat tissues. Therefore, we postulate that ceramide accumulation that accompanies obesity affects adipocytokine secretion.

## Figures and Tables

**Table 1:** Metabolic parameters in rats with HFD-induced insulin resistance and under myriocin (Myr) treatment.

Metabolic Parameter	Control	HFD	HFD/Myr
Fasting plasma glucose concentration [mg/dL]	97.8 ± 14.3	139.2 ± 27.1 ^a^	86.6 ± 19.9 *
Fasting insulin concentration [µU/mL]	51.7 ± 12.9	65.9 ± 9.6	55.8 ± 10.0
HOMA-IR	2.08 ± 0.6	3.75 ± 0.84 ^a^	1.96 ± 0.52 *
Plasma FFA concentration [µmol/L]	279.1 ± 34.7	362.2 ± 24.4 ^a^	403.3 ± 49.3 *
Plasma adiponectin concentration [µg/mL]	18.9 ± 2.8	10.8 ± 1.6 ^a^	22.9 ± 3.1 *
Plasma TNF-α concentration [ng/mL]	1.52 ± 0.19	2.57 ± 0.4 ^a^	1.41 ± 0.40 *

^a^—vs. Control; *—vs. HFD.

**Table 2:** Plasma FFA concentration in rats with HFD-induced insulin resistance and under myriocin (Myr) treatment. Values are mean (µmol/L of plasma) +/− SD.

Fatty Acid	Control	HFD	HFD/Myr
C14:0	13.2 ± 2.6	14.8 ± 2.6	15.7 ± 0.7
C16:1	7.3 ± 1.4	2.0 ± 0.3 ^a^	2.5 ± 0.4 ^a^
C18:2	64.9 ± 12.8	59.9 ± 3.2	102.8 ± 13.9
C16:0	61.2 ± 8.7	60.7 ± 4.8	83.4 ± 13.5 *
C18:1	66.2 ± 14.2	45.6 ± 8.7 ^a^	68.7 ± 11.6 *
C18:0	36.6 ± 4.1	91.4 ± 10.5 ^a^	94.1 ± 15.3 *
C20:0	1.02 ± 0.2	4.3 ± 0.9 ^a^	3.1 ± 0.3 ^a^^,^*
C22:0	4.5 ± 0.7	11.7 ± 0.9 ^a^	15.0 ± 2.4 ^a^
C24:1	1.7 ± 0.4	2.3 ± 0.5	11.0 ± 0.4 ^a,^*
C24:0	22.5 ± 3.0	69.4 ± 15.6 ^a^	6.8 ± 0.4 *

^a^—vs. Control; *—vs. HFD.

**Table 3:** Ceramide in two depots of adipose tissue. Values are mean (pmol/mg tissue) +/− SD.

	Visceral Fat Tissue			Subcutaneous Fat Tissue		
Ceramide	Control	HFD	HFD/Myr	Control	HFD	HFD/Myr
C14-Cer	0.007 ± 0.001	0.014 ± 0.003 ^a^	0.003 ± 0.0005 ^a,^*	0.03 ± 0.006	0.04 ± 0.006 ^a^	0.01 ± 0.000 ^a,^*
C16-Cer	1.05 ± 0.19	1.82 ± 0.30 ^a^	1.36 ± 0.27 ^a,^*	2.4 ± 0.5	4.1 ± 0.6 ^a^	1.68 ± 0.37 ^a,^*
C18:1-Cer	0.008 ± 0.001	0.03 ± 0.006 ^a^	0.01 ± 0.002 ^a,^*	0.03 ± 0.01	0.08 ± 0.02 ^a^	0.03 ± 0.003 *
C18-Cer	0.10 ± 0.02	0.56 ± 0.10 ^a^	0.20 ± 0.04 ^a,^*	0.74 ± 0.07	1.22 ± 0.18 ^a^	0.43 ± 0.06 ^a,^*
C20-Cer	0.09 ± 0.018	0.34 ± 0.09 ^a^	0.09 ± 0.01 ^a,^*	0.37 ± 0.10	0.58 ± 0.05 ^a^	0.15 ± 0.02 ^a,^*
C22-Cer	0.52 ± 0.08	0.63 ± 0.09 ^a^	0.22 ± 0.03 ^a,^*	0.65 ± 0.10	1.32 ± 0.3 ^a^	0.41 ± 0.06 ^a,^*
C24:1-Cer	0.53 ± 0.11	0.63 ± 0.09 ^a^	0.38 ± 0.06 ^a,^*	0.91 ± 0.16	1.27 ± 0.3 ^a^	0.74 ± 0.08 ^a,^*
C24-Cer	2.02 ± 0.39	2.9 ± 0.34 ^a^	0.56 ± 0.09 ^a,^*	1.72 ± 0.23	4.04 ± 0.64 ^a^	1.05 ± 0.15 ^a,^*
Total Cer	4.35 ± 0.61	6.94 ± 0.85 ^a^	2.84 ± 0.35 ^a,^*	6.83 ± 0.80	12.71 ± 1.0 ^a^	4.50 ± 0.52 ^a,^*

^a^—vs. Control; *—vs. HFD.

**Table 4:** DAG in two depots of adipose tissue. Values are mean (pmol/mg tissue) +/− SD.

	Visceral Fat Tissue			Subcutaneous Fat Tissue		
Diacylglycerol	Control	HFD	HFD/Myr	Control	HFD	HFD/Myr
16/16	0.41 ± 0.07	1.09 ± 0.18	0.6 ± 0.06 *	1.07 ± 0.27	3.45 ± 0.64	3.07 ± 0.43 *
16/18:1	1.8 ± 0.23	7.1 ± 1.0	5.3 ± 0.67 ^a,^*	5.20 ± 0.78	19.4 ± 3.2 ^a^	11.0 ± 0.7 ^a,^*
16/18:2	1.08 ± 0.19	3.09 ± 0.45 ^a^	2.13 ± 0.22 *	2.0 ± 0.5	6.33 ± 0.97 ^a^	4.05 ± 0.33 ^a,^*
16/18	0.28 ± 0.05	1.09 ± 0.18 ^a^	0.55 ± 0.08 *	0.93 ± 0.11	3.1 ± 0.68 ^a^	2.14 ± 0.16 ^a,^*
18:1/18:1	1.14 ± 0.25	6.0 ± 1.02 ^a^	3.19 ± 0.43 *	3.39 ± 0.39	10.9 ± 1.3 ^a^	6.08 ± 0.30 *
18:1/18:2	1.1 ± 0.20	5.32 ± 0.85 ^a^	2.52 ± 0.27 *	2.18 ± 0.63	14.9 ± 2.1 ^a^	6.92 ± 0.44 ^a,^*
18/18:1	0.01 ± 0.004	0.05 ± 0.008 ^a^	0.027 ± 0.005 ^a,^*	0.08 ± 0.008	0.35 ± 0.049 ^a^	0.12 ± 0.02 ^a,^*
Total DAG	5.84 ± 0.47	23.73 ± 1.35 ^a^	14.32 ± 1.1 ^a,^*	14.88 ± 1.31	58.57 ± 4.28 ^a^	33.46 ± 1.46 ^a,^*

^a^— vs. Control; *— vs. HFD.

## Abbreviations

Cer	Ceramide
DAG	Diacylglycerols
HFD	High fat diet
IRes	Insulin resistance
OGTT	Oral glucose tolerance test
ITT	Insulin tolerance test
HSL	Hormone sensitive lipase
HOMA-IR	Homeostatic Model Assessment for Insulin Resistance
LC/MS/MS	Liquid chromatography tandem mass spectrometry
VAT	Visceral adipose tissue
SAT	Subcutaneous adipose tissue
FFA	Free fatty acids
